# Anti-*Leishmania major* Properties of *Nuphar lutea* (Yellow Water Lily) Leaf Extracts and Purified 6,6′ Dihydroxythiobinupharidine (DTBN)

**DOI:** 10.3390/pathogens13050384

**Published:** 2024-05-06

**Authors:** Orit Shmuel, Aviv Rasti, Melodie Zaknoun, Nadav Astman, Avi Golan-Goldhirsh, Orly Sagi, Jacob Gopas

**Affiliations:** 1 Shraga Segal Dept. of Microbiology Immunology and Genetics, Faculty of Health Sciences, Ben Gurion University of the Negev, Beer Sheva 8410501, Israel; oritsh194@gmail.com (O.S.); avivra@clalit.org.il (A.R.); orlisa@clalit.org.il (O.S.); 2 Department of Clinical Biochemistry & Pharmacology, Faculty of Health Sciences, Ben-Gurion University of the Negev, Beer Sheva 8410501, Israel; melodyzaknoun@gmail.com; 3 Department of Dermatology, Sheba Medical Center, Sackler Faculty of Medicine, Tel Aviv University, Tel-Hashomer, Tel Aviv 39040, Israel; nadav.astman@sheba.health.gov.il; 4 The Jacob Blaustein Institutes for Desert Research (BIDR), French Associates Institute for Agriculture and Biotechnology of Drylands, Ben-Gurion University of the Negev, Sede Boqer Campus, Beer Sheva 8410501, Israel; avigolan@bgu.ac.il; 5 Laboratory of Microbiology, Soroka University Medical Center, Beer Sheva 84101, Israel

**Keywords:** *Leishmania major*, cutaneous leishmaniasis, 6,6′-dihydroxythiobinupharidine (DTBN), *Nuphar lutea*, anti-*Leishmania* small molecule

## Abstract

Cutaneous leishmaniasis (CL) is a zoonotic disease, manifested as chronic ulcers, potentially leaving unattractive scars. There is no preventive vaccination or optimal medication against leishmaniasis. Chemotherapy generally depends upon a small group of compounds, each with its own efficacy, toxicity, and rate of drug resistance. To date, no standardized, simple, safe, and highly effective regimen for treating CL exists. Therefore, there is an urgent need to develop new optimal medication for this disease. Sesquiterpen thio-alkaloids constitute a group of plant secondary metabolites that bear great potential for medicinal uses. The nupharidines found in *Nuphar lutea* belong to this group of compounds. We have previously published that *Nuphar lutea* semi-purified extract containing major components of nupharidines has strong anti-leishmanial activity in vitro. Here, we present in vivo data on the therapeutic benefit of the extract against *Leishmania major* (*L. major*) in infected mice. We also expanded these observations by establishing the therapeutic effect of the extract-purified nupharidine 6,6′-dihydroxythiobinupharidine (DTBN) in vitro against promastigotes and intracellular amastigotes as well as in vivo in *L. major*-infected mice. The results suggest that this novel anti-parasitic small molecule has the potential to be further developed against *Leishmania.*

## 1. Introduction

Leishmaniasis is a disease caused by an obligate intracellular parasite and transmitted by a female sandfly’s bite [1]. The disease is distributed worldwide [2] and is considered as a conspicuous global cause of death by infectious diseases by the World Health Organization (WHO) [3]. Leishmaniasis is categorized not only by the geographic region but also by the parasite strain, which affects the severity, form, and symptoms of the disease [4].

Parasites are deposited into the mammalian host’s skin during the blood meal of infected female phlebotomine sand flies. The parasite has a digenetic life cycle alternating between sandfly vectors and mammalian hosts, adapting to changing environments via life cycle-specific expression of genes. It has two main morphologic life stages: amastigote, the intracellular shape, and promastigote, the sandfly’s form. Amastigote is the obligatory intracellular parasite in the animal reservoir or humans. It is elliptic and nonmotile and it develops mainly in the host’s macrophages. Promastigotes develop in the sandfly midgut. They are motile, extracellular, long, and narrow, with long flagella. At least twenty different *Leishmania* species can infect humans causing three main clinical manifestations, including cutaneous, mucocutaneous, and visceral forms. The outcome of the disease depends on the infecting species along with host factors. Cutaneous leishmaniasis (CL) is the most common form characterized by the appearance of a skin ulcer at the sand fly bite site, usually on exposed body parts. Although skin lesions are most of the time self-healing and localized, they can leave seriously disfiguring and disabling life-long scars. Mucocutaneous leishmaniasis (MCL) is a major CL complication that can manifest days to years following the cutaneous lesion. MCL develops when the parasites migrate from the localized skin lesion to mucosal tissues of the nose, mouth, and throat cavities, through lymphatics and blood vessels. This can lead to extensive destruction of the oral or nasal mucosa and potentially become life-threatening. Visceral leishmaniasis (VL) is the deadliest form of the disease resulting from parasite dissemination from the skin to visceral organs, such as the spleen and the liver. This leads to organ dysfunction, fever, and weight loss, and is usually fatal if left untreated [5].

Currently, leishmaniasis treatment is a challenge worldwide. Treatment failure is an increasing problem due to the development of resistant parasites to most drugs. No vaccine is available for humans. Chemotherapies are based on drugs with variable efficacy and toxicity related to the length of therapeutic schemes and high doses. The number of effective drugs available against the disease (i.e., sodium stibogluconate, pentamidine, amphotericin B, miltefosine, paromomycin) is limited [6,7] and often selected for drug-resistant strains [8]. Thus, safe and therapeutically useful anti-leishmanial compounds are needed. Natural products from a variety of sources, some based on ethnomedicinal traditions, are an important source for anti-parasite drug development.

We have previously published the ability of an alkaloid semi-purified mixture extracted from the aquatic plant *Nuphar lutea, which consists mainly of* dimeric sesquiterpene thioalkaloids called thiobinupharidines and thiobinuphlutidines [9], that is effective in vitro against free as well as intracellular *Leishmania major* (*Lm*) parasites [10,11,12].

The extract from *Nuphar lutea* and its extract-purified molecule 6,6′-dihydroxythiobinupharidine (DTBN) are pleiotropic in their action. The semi-purified extract has anti-inflammatory activity, it downregulates NF-κB and partially prevents LPS-induced septic shock and peritonitis [9,13]. The extract is also anti-metastatic and is synergistic with conventional chemotherapy drugs both in vitro and in vivo and it activates phospho-extracellular signal-regulated kinases (ERKs) [14]. The extract is also anti-viral against the measles virus [15]. We have also published a variety of other DTBN effects [16,17,18,19,20,21]. It’s properties and their relevance to treatment of *Leishmania* are discussed below.

Based on the effectiveness of the *Nuphar lutea* extract against both leishmanial promastigotes and amastigotes in vitro, we present here data on the extract’s efficacy against *L. major*-infected mice as well as the efficacy of DTBN in vitro and in vivo. The in vivo results represent proof of concept of the potential of this molecule as an anti-leishmanial and possibly as a wider anti-parasitic molecule to be further investigated to be used in treatment.

## 2. Materials and Methods

### 2.1. L. major Promastigote Culture

Promastigotes were obtained from the parasitology clinical laboratory of the Soroka University Medical Center, Beer Sheva, Israel. The parasites were grown as extracellular promastigotes in 75 cm^2^ flasks and maintained in an incubator at 28 °C. RPMI medium + 10% FCS and 1% pen-strep were added to the culture twice a week. In addition, 10 mL was taken out from the flask and 10 mL of growth medium was added to the flask.

### 2.2. The Survival Rate of L. major Promastigotes Treated with DTBN

**Figure 1:**
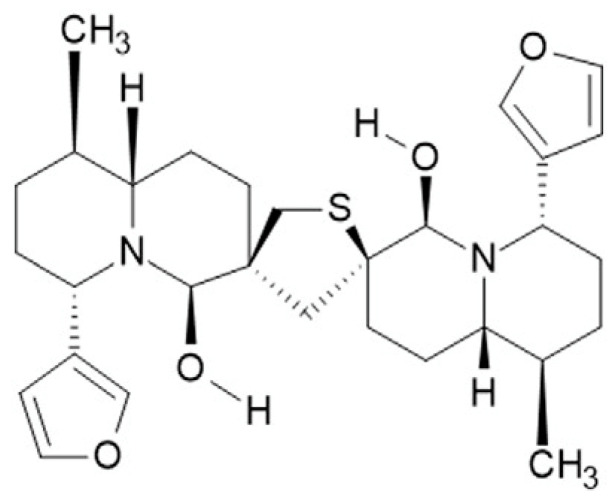
Structure of 6,6′-dihydroxythiobinupharidine (DTBN).

We have previously described the ability of the semi-purified extract of *Nuphar lutea* to inhibit free promastigotes and macrophage intracellular amastigotes. To test the ability of the purified molecule 6,6′-dihydroxythiobinupharidine (DTBN) (Sigma/Merck, St. Louis, MO, USA. Cat. SMB00609) (Figure 1) to inhibit the promastigotes, 11 × 10^6^/well *L. major* promastigotes were added to 96-well plates. Each well was treated with paromomycin (Paro.) (Sigma/Merck, MO, USA) as the gold-standard, DTBN 0.1 µg/mL 0.2 µg/mL or a vehicle. After 48 h, the motile live promastigotes were counted, out of 100 parasites in triplicate. Their viability was confirmed by trypan blue on a hemocytomer. The experiment was repeated 3 times.

### 2.3. Macrophage Isolation from C3H Mice

Three days before the in vitro experiment began, 3 mL of thioglycolate (Sigma/Merck, MO, USA) was injected IP into two 8-week-old C3H male mice. Three days later, mice were asphyxiated by CO_2_, and peritoneal macrophages were obtained by washing the peritoneal cavity with 10 mL sterile PBS. Cells were centrifuged at 1000 RPM for 5 min and counted.

### 2.4. Macrophage Preparation, Infection with Leishmania Major, and Treatments

First, 3 × 10^5^ macrophages in 1 mL RPMI complete medium were seeded in each well of a 24-well plate. Each well contained a sterile glass coverslip for cell attachment. Three hours after macrophage attachment, they were washed twice with PBS, and promastigotes were added, i.e., 1 × 10^6^ in 1 mL of complete RPMI medium. Then, 24 h after cell infection, DTBN 2,3 or 5 µg/mL or growth medium was added. After 72 h, the wells were washed twice with PBS and fixed with methanol for 40 min, dried and 1:10 Giemsa (Sigma/Merck, MO, USA) in double-distilled water (DDW) was added for 20 min. The wells were then washed with water and dried on Whatman paper. Following staining, the slides were removed from the wells and glued with Hydromount to a regular glass slide. In parallel, 72 h later, equally treated macrophage cultures in triplicate were collected by scraping, DNA was extracted by Seegene Nimbus automatic PCR and cDNA was synthesized. The DNA samples were run in multiplex hydrolysis probe-based real-time PCR (mqRT-PCR) with primers 6-FAM-5′-TCTCT/ZEN/ and CCCTCCCGCCAAAAACC/3labFQ-3′. The primers were common to all *Leishmania* for the ITS (*Leishmania* internal transcribed spacer 1, 5.8S ribosomal RNA gene) conserved region. The experiment was repeated twice.

Multiplex PCR is the simultaneous detection of multiple targets in a single reaction well, with a different pair of primers for each target. This technique requires two or more probes that can be distinguished from each other and detected simultaneously, with each probe labeled with a differently colored fluorophore.

*L. major* cDNA relative amounts were calculated based on a calibration curve on cDNA derived from promastigotes grown in culture and counted. Serial dilutions were prepared and tested in triplicate by multiplex qPCR. The procedure and validation of the primers are detailed in the work of Sagi et al., 2017 [22].

### 2.5. Preparation of Promastigotes for Electron Microscopy

Promastigotes were prepared by conventional TEM and SEM techniques. For TEM, the promastigote suspensions of untreated or treated with 0.1 µg/mL DTBN for 24 h were washed in PBS three times for 5 min, and fixed in 0.2% gluteraldehyde in 0.05 M cacodylate buffer (pH 7.2) for 3 min at room temperature. The collected cells were fixed again with 2% gluteraldehyde in 0.05 M cacodylate buffer at 4 °C for 60 min. After 3 washes in 0.05 M cacodylate buffer, the cell pellet was post-fixed in 1% OsO_4_ in cacodylate buffer at 4 °C for 60 min, and washed twice for 10 min in cacodylate buffer, dehydrated in graded ethanol concentrations, and embedded in an Araldite mixture. Thin sections were stained in uranyl acetate for 15 min and observed using a transmission electron microscope, JEM 2100F.

For SEM, the promastigote suspensions were treated in the same manner as for TEM and centrifuged. The supernatants were then removed, and the pellets were re-suspended into 500 μL of PBS. This centrifuge-washing step was repeated twice. Following the removal of the PBS supernatant, 500 μL of 2.5 % glutaraldehyde in PBS solution was added, and the pellets were re-suspended and incubated at 4 °C for at least 4 h, after which the suspension was centrifuged and washed with 500 μL of ultrapure water. Dehydration was then performed by adding 30% (*v*/*v*) ethanol solution and carrying out immersion for 15 min at 4 °C, followed by centrifugation at 10,000 rpm for 5 min and the removal of the supernatant. This stepwise dehydration was subsequently carried out for 50, 70, and 100% ethanol. Before imaging, the dehydrated cell suspension was vortexed and 10 μL was spread onto an ethanol-cleaned silicon substrate; this was followed by air-drying inside a biosafety hood. A platinum layer was then used to sputter-coat the dry sample under 20 mA current for 20 s to reduce the charging effect. A Zeiss Supra55VP FESEM was used for imaging of the samples.

### 2.6. In Vivo Experiments

Treatment reagents

Pentostam, sodium stibogluconate (Sigma/Merck, MO, USA), a known drug against *Leishmania* was used as the gold standard. The stock concentration was 100 mg/mL. Mice were injected with Pentostam (20 mg/kg/mouse intraperitoneally) [23].

*Nuphar lutea* semi-purified extract (NUP.E) of *Nuphar lutea* leaves was prepared as described in [9]. The stock concentration of NUP.E. was 10 mg/mL. Mice were treated IP with NUP.E 20 mg/kg/mouse in 100 µL (about 400 µg per mouse).

Acidic double-distilled water (DDW + HCl 0.05N) was used as the NUP.E solvent. Mice were treated with NUP.E’s vehicle intraperitoneally. The in vitro anti-leishmanial effect of the extract has been previously demonstrated [10,11]. Here, we studied its effect in vivo on *Leishmania major* (*Lm*)-infected mice as well as the in vivo effect of DTBN.

Purified 6,6′-dihydroxythiobinupharidine (DTBN) was also used. The stock concentration of DTBN was prepared in 1 mg/mL of DMSO. Mice received intralesional treatment with 20 µg/40 µL DMSO.

Six-week-old male Balb/c mice were purchased from Envigo RMS (Israel) and kept in the animal facility of the Faculty of Health Sciences, Ben-Gurion University of the Negev. Animal experiments protocols were approved by the University Animal Experiments Committee (IL-98-12-2019).

Mice were anesthetized with 5% and then 3% isoflurane for 2–5 min before shaving, *L. major* injection, or photography of lesions.

#### 2.6.1. Lm Injection

One day before infection, the mice were shaved in the tail base area with an electrical shaver. Then, NAIR commercial hair-removing cream with potassium thioglycolate as an active ingredient was applied for 30 s. The cream was removed with a gauze pad and Septol. Then, 1 × 10^8^/100 µL *L. major* promastigotes were inoculated subcutaneously per mouse to the tail base. About 10 days after *L. major* injection, wounds appeared and were photographed as the first time-point. When the wounds appeared, treatment was started. Two different experiments were performed.

#### 2.6.2. Intraperitoneal Treatment

In total, 20 mice per group were treated intraperitoneally (IP) with 20 mg/kg/mouse in 100 µL of NUP.E. or a vehicle (HCl 0.05N) once a day for 10 days. Pictures were taken pretreatment, after 8 days of treatment, 5 (day 13) and 30 (day 28) days after the last treatment.

#### 2.6.3. Intralesional Treatment

In total, 18 mice per group received intralesional (IL) treatment with 20 µg of DTBN in 40 µL of the vehicle (DMSO) or Pentostam 20 mg/kg/mouse intraperitoneally once a day for 15 days (R1). Treatments were then stopped for 12 days and restarted for 7 more days (R2). A wound area > 1.0 cm^2^ is considered a large wound area; therefore, the animals were removed from the experiment if they displayed a wound area of this size. The leishmanial wounds were measured by using an image analysis program (Digimizer MedCalc Software Ltd. Ostend, Belgium). This is a flexible image analysis software package that allows precise manual measurements as well as automatic object detection with measurements of object characteristics.

The pictures obtained include X-rays, micrographs, etc. The images can be rotated, flipped or straightened. The image brightness and contrast can be adjusted [24].

## 3. Results

### 3.1. Survival of DTBN-Treated Lm Promastigotes

**Figure 2:**
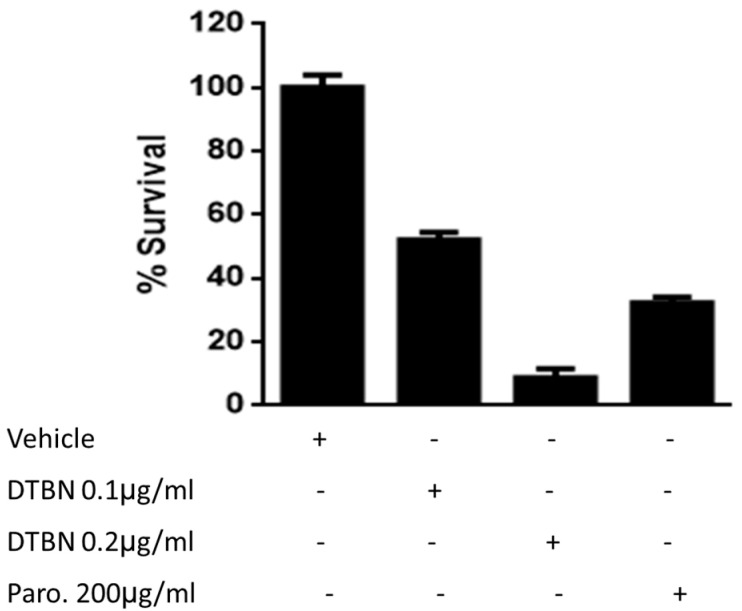
Survival of DTBN-treated *Lm* promastigotes. First, 1 × 10^6^/well *Leishmania major* promastigotes in 1 mL were added to 96-well plates in a 200 µL final volume (200,000 parasites/well). Each well was treated with paromomycin (Paro.) as the gold standard, DTBN 0.1 µg/mL or DTBN 0.2 µg/mL or vehicle. Then, 48 h later, the motile (live) promastigotes were counted out of 100 parasites and the percentage of viable parasites was confirmed on a hemocytometer with trypan blue. The surviving promastigotes are presented as a percentage of untreated live promastigotes. Mean and SD were calculated.

The effect of DTBN on the viability of promastigotes was determined. The results in Figure 2 show that DTBN has a toxic dose–response effect of about a thousand-fold lower concentration than paromomycin.

### 3.2. Transmission (TEM) and Scanning Electron Microscopy (SEM) of Lm Promastigotes Treated with DTBN

**Figure 3:**
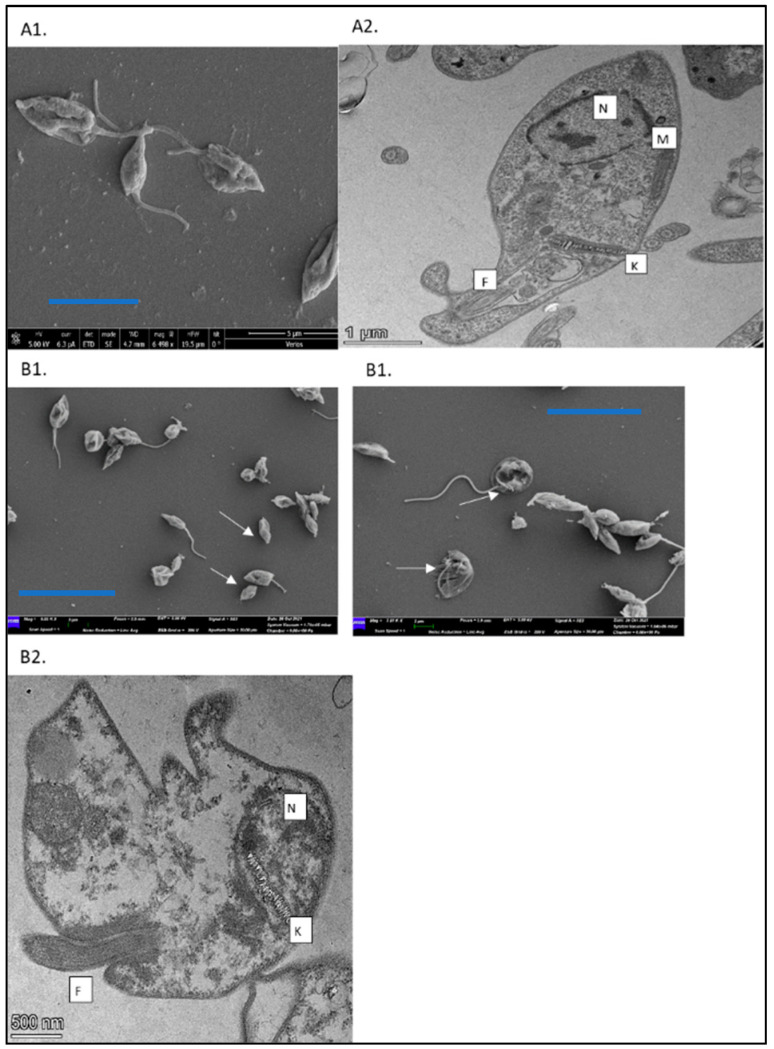
Transmission (TEM) and scanning electron microscopy (SEM) images of *L. major* promastigotes treated with DTBN. *L. major* promastigotes were treated with growth media or 0.1 µg/mL DTBN for 24 h. The cells were then washed with PBS and processed as described for EM evaluation. (**A1**) Control, untreated promastigotes with SEM pictures (bar 5 mm), (**A2**) Untreated promastigotes, with TEM pictures. (**B1**) SEM pictures of treated promastigotes at two magnifications (**left B1**) (5.55K (bar 15 mm) and (**right B1**) 7.5K (bar 7.5 mm) magnification (bar 7.5 mm)). Arrows show damaged promastigotes, some lack flagella or show cell body and organelle damage. (**B2**) TEM of promastigotes treated with 0.1 µg/mL DTBN. M—mitochondria; N—nucleus; F—flagella; K—kinetoplast.

The TEM and SEM pictures of treated promastigotes shown in Figure 3 confirm the reduced viability as a result of treatment with DTBN. Damaged promastigotes are shown by both techniques, some lacking normal cell morphology and lack of flagella as well as organelle damage.

Control parasites possessed typical slender cell bodies with smooth cell surfaces and elongated flagella. Treated promastigotes (0.1 mg/mL of DTBN) exhibited shrunken morphology showing signs of multiseptation, indicating a possible loss of cell volume when treated. Among the population of promastigotes being observed at this DTBN concentration, about 70% showed altered morphology and lack of flagellum.

### 3.3. DTBN Treatment of Lm-Infected Macrophages Reduces the Number of Intracellular Amastigotes

**Figure 4:**
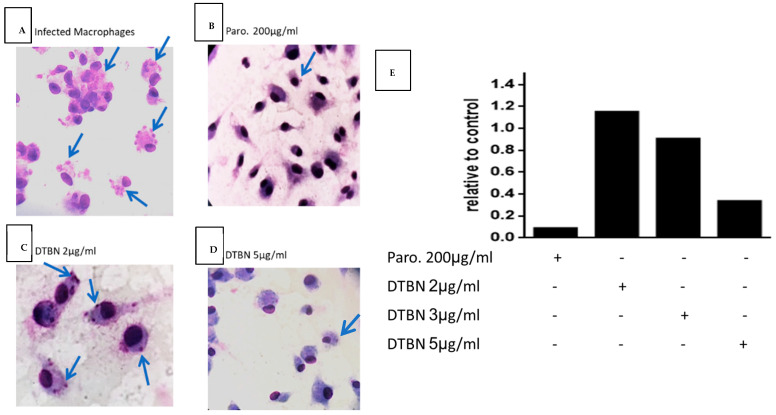
DTBN treatment of infected macrophages reduces the amount of intracellular *L. major* amastigotes. First, 24-well plates were covered with slides. Then, 3 × 10^5^/well C3H peritoneal macrophages were left to adhere for 3 h on glass-slide-covered wells. Next, 1 × 10^6^/well *L. major* promastigotes were added to each well (in triplicate). After 24 h, cells were treated (**A**) vehicle, (**B**) with paromomycin (Paro) 200 µg/mL, (**C**) DTBN 2 µg/mL or (**D**) DTBN 5 µg/mL. After 72 h, the slides were Giemsa stained and the intracellular amastigotes are shown by the arrow. Pictures were taken at a ×1000 magnification. (**E**) In parallel, 72 h later, equally treated macrophage cultures were collected, and cDNA was synthesized. The DNA samples were run in multiplex hydrolysis probe-based real-time PCR (mqRT-PCR). *L. major* cDNA relative amounts as compared to the positive control paromomycine were calculated based on a promastigote DNA calibration curve. The results were normalized to vehicle-treated values. The *Leishmania*-infected macrophage cDNA sample value was 1. Thus, 2 µg/mL was not effective and 3 and 5 µg/mL showed a dose response of about 10% and 70% effectiveness.

The results in Figure 4 show that non-toxic DTBN concentrations inhibit in a dose–response manner the presence of intracellular amastigotes in macrophages. Interestingly, the concentrations of DTBN required to kill amastigotes are ten-fold higher than those required to kill free promastigotes. Thus, promastigotes and amastigotes are killed at DTBN concentrations that do not affect the host macrophages. These results are of relevant therapeutic importance.

### 3.4. Intraperitoneal Injection of NUP.E Decreases Leishmanial Wound Size in Mice

**Figure 5:**
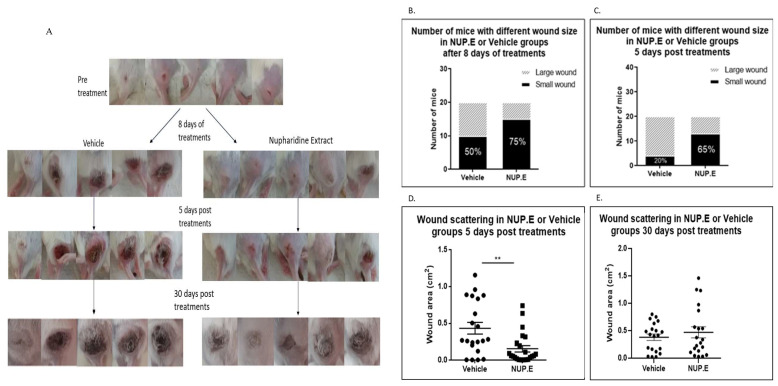
*Nuphar lutea* semi-purified extract (NUP.E) reduces the size of *Lm* wounds in treated mice. First, 1 × 10^8^/100 µL *L. major* promastigotes were injected into the tail base of 40 Balb/c male mice. Ten days later, following the appearance of the wound, 20 mg/kg/mouse NUP.E or acidic water (vehicle) was IP injected daily into mice for 8 days. Small wounds were defined as <0.1 cm^2^; large wounds as >0.1 cm^2^. The wound size was determined by the Digimizer program. Pictures of the wounds were taken at days 0 and 8 after treatment as well as 5 and 30 days post-treatment. (**A**) Representative pictures of differences in wound size between treated and control groups. (**B**) Proportion of mice with small/large wound area after 8 days of treatment. (**C**) Proportion of mice with small/large wound area 5 days post-treatment. (**D**) Wound area scattering of treated mice (each dot represents the wound area of one mouse in the experiment) as compared to control, 5 days post-treatment (*t*-test; *p* < 0.0016, ** *p* < 0.01). (**E**) Wound area scattering of treated mice as compared to control, 30 days post-treatment. No significant difference in wound size was observed between the two groups. Mice were sacrificed when the wound area reached 1.5 cm^2^.

Based on the effectiveness of NUP.E against *Lm* promastigotes in vitro, we established as proof of concept a mouse model to show its effect in vivo. Ten days after parasite inoculation and the appearance of a lesion in the tail base, NUP.E or the vehicle were injected IP once a day for 8 days. Wound size was measured at different time points. The results in Figure 5 show that IP treatment of NUP.E reduced the size of the lesions. This effect also continued for 5 days after the treatment was discontinued. In addition, 30 days post-treatment, the curative effect of NUP.E was not apparent anymore and there was no difference in wound size between the treated and control mice.

### 3.5. Intra-Lesion Injection of Purified DTBN Reduces Wound Size and Partially Cures L. major Infected Mice

**Figure 6:**
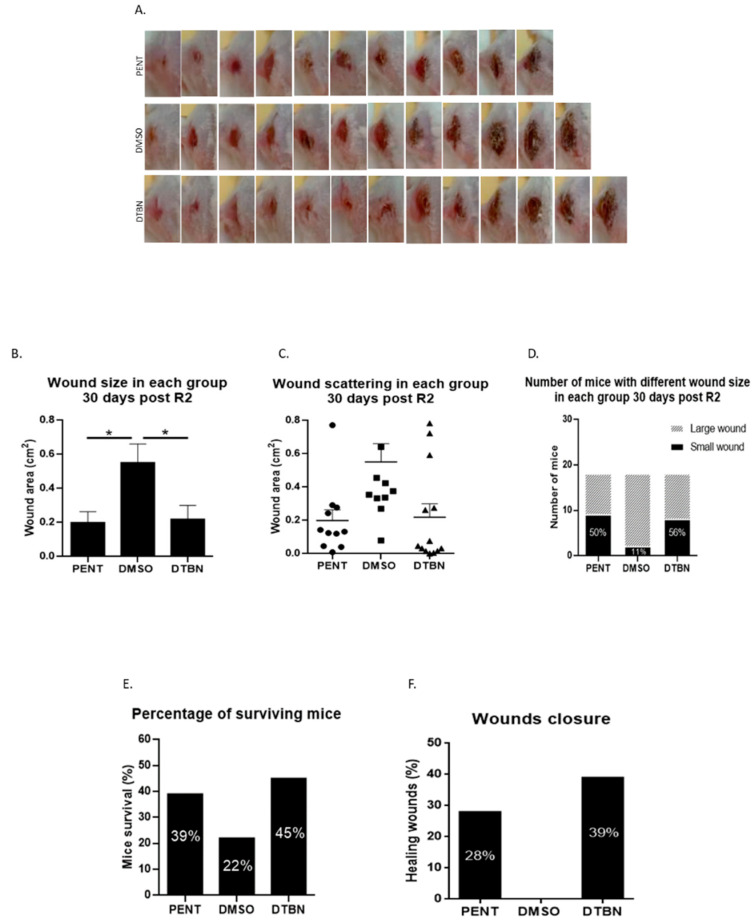
Reduction in in vivo wound size of *L. major*-infected mice by intra-lesion (IL) injection of DTBN or vehicle. First, 1 × 10^8^/100 µL *L. major* promastigotes were injected into the mouse tail base. Next, 10 days afterward, the lesions appeared and 20 µg/40 µL of DTBN or vehicle (diluted DMSO) (n = 18) was IL injected. As a positive control, 20 mg/kg/mouse Pentostam (PENT) (n = 18) was IP injected. All treatments were given once a day for 15 days (R1) and the treatment was then stopped for 12 days and resumed for 7 more days (R2). Wound pictures were taken 30 days post-R2. (**A**) Increasing wound size from the smallest to the largest in each group, 30 days post-R2. (**B**) Wound size 30 days post-R2 of mice treated with DTBN or Pentostam as compared to the vehicle group. (Mean and SEM were calculated by Anova test; *p* < 0.0128, * *p* < 0.05). (**C**) Wound size distribution 30 days post-R2. (**D**) Proportion of mice with large/small wounds 30 days post-R2. The number of mice in each group was 18 (small wound: cm^2^ < 0.1; big wound: cm^2^ > 0.1 The wound size was analyzed by the Digimizer program). (**E**) The proportion of mice who survived in each group (18 mice/group) was determined at the endpoint of the experiment, 48 days post-R2. Mice were sacrificed when the wound area reached 1.5 cm^2^. (**F**) Wound size was compared at 48 days post-R2 to the wound size at 30 days post-R2. Unchanged wound size or size reduction was considered a cured wound.

Following positive in vivo results in mice treated with NUP.E, we then decided to treat the mice with purified DTBN via intralesional (IL) injection. *Lm* lesions were induced as described above and treatment started when the wounds appeared, 10 days after the parasite injection. DTBN or the vehicle (DMSO) was injected IL. The gold-standard positive control treatment, Pentostam (PENT), was injected intraperitoneally as described in the literature [23]. Mice were injected once a day for 15 days (R1). After 15 days of treatment, the treatment stopped for 12 days and resumed for 7 more days (R2). Wound size was measured at different time points. The long-term results, 30 days post-R2, show that IL treatment significantly decreases the size of the lesion and is comparable to mice treated with Pentostam, including better wound closure in DTBN-treated mice (Figure 6).

## 4. Discussion

NUP.E has been previously investigated in our laboratory and was found to have in vitro significant anti-leishmanial activity against both promastigotes and amastigotes [9]. Here, we focused on establishing an in vivo model to examine the therapeutic potential of both NUP.E and DTBN. First, we determined that DTBN, similarly to the extract, significantly eliminates both amastigotes and promastigotes. The extracellular promastigotes are about 10-fold more sensitive than the intracellular amastigotes in infected macrophages (0.2 mg/mL in Figure 2 and 3.0 mg/mL in Figure 4, respectively). This difference is not surprising since DTBN directly reaches the promastigote and for DTBN to be effective intracellularly, it first has to be internalized into the macrophage to avoid possible inactivation and then reach the parasite, requiring a higher effective dose.

TEM and SEM pictures confirmed the reduction in viable amastigotes and showed damaged cells.

The next step was to establish the in vivo model. We first tested the effect of the extract. The infected mice were treated with IP injections [25], NUP.E or vehicle once a day for eight days. The results presented in Figure 3 exhibit a significant reduction in the *L. major* wound area in the NUP.E group as compared to the control after 8 days of treatment, which was also apparent 5 days post-treatment. We followed the mice for 30 days. The lesions that initially did not grow due to the treatment eventually increased in area 30 days after the treatment was stopped. This experiment is proof of concept for the effectiveness of the extract and suggests that the treatment protocol should be optimized and improved by longer-term treatment and higher non-toxic concentrations to achieve comprehensive parasite elimination and complete wound closure. The *L. major* wound size generally correlates with the parasite burden in the infected mouse as shown in previous studies [26,27]; therefore, measuring wound size is an accepted way of quantifying therapeutic effectiveness.

Following the positive in vivo results with NUP.E, we decided to treat infected mice with the purified molecule, DTBN, by intralesional injection (IL), another accepted treatment modality in the clinic [25]. As controls, IL injection of the vehicle DMSO, or Pentostam (IP), the positive gold-standard treatment in mice, was carried out [28]. The experience gained in our previous in vivo experiments suggested we should treat the mice for longer periods. Thus, two treatment cycles were performed. Mice were injected once a day for 15 days (R1). After 15 days of treatment, the treatment stopped for 12 days and resumed for 7 more days (R2). The results shown in Figure 6 demonstrate a significant reduction in wound size in the DTBN group, 30 days post-R2, with 39% of the wounds markedly improved with complete closure and healing. None of the DMSO-treated mice recovered. Interestingly, the Pentostam treatment was less effective than DTBN, healing only 28% of the mice [23,29]. It seems that both NUP.E IP and DTBN IL treatments are only partially effective. The treatment protocol for both modalities must be optimized by a higher concentration, length of treatment, or more cycles of treatments. To determine the acceptable non-toxic concentration of NUP.E and DTBN, toxicology and pharmacodynamics experiments should be carried out, and the best treatment modality determined. Ointment or oral treatment should also be investigated.

A variety of molecular drug targets against *Leishmania* have been described. Although we have not specifically tested the extract or DTBN directly on *Leishmania* target molecules, we have evidence that they act on similar molecules in other cell types. We provide a short, albeit indicative description of their action to support our work.

*L. major* induces innate immunity and inflammation by mast cell stimulation and by secreting pro-inflammatory mediators by these cells. Reactive oxygen species (ROS) or nitric oxide (NO) are produced during an inflammation response that leads to oxidative damage [30]. We observed that NUP.E modulates NF-kB and increases the production of inducible nitric oxide synthase (iNOS), NO production, and killing of intracellular amastigotes [10]. We showed that DTBN acts as an oxidative agent, it induces elevation of ROS cytosolic levels, decreases glutathione levels and induces apoptosis by caspase activation in acute myelocytic leukemia cells. The DTBN toxicity range was determined in these leukemic cell lines [17]. These combined properties are effective against free and intracellular parasites in vitro and in vivo. Since DTBN has multiple targets, its combined, pleiotropic effects on cells make it an effective therapeutic agent by itself or in combination with established treatment modalities.

Neutrophils are rapidly recruited to the skin following *Leishmania* infection and they play a critical role in phagocytosing the parasites and in shaping the immune response [31,32]. We have previously published that DTBN primes neutrophils against bacteria present in gum inflammation, and enhances phagocytosis, ROS production, and extracellular trap (NET) formation, as NETs are made of a network of extracellular strings of DNA that bind to pathogenic microbes [16].

Our results are consistent with the ability of DTBN to activate neutrophils, inducing ROS, and support our results of limiting infection with *L.m*.

DTBN very efficiently and covalently inhibits human type II topoisomerase [18] and conventional Protein Kinase Cs (PKC), especially PKC alpha and PKC gamma [19]. DTBN also inhibits cysteine proteases such as cathepsins S, B, L, and papain [20]. Recently, we published that DTBN inhibits an additional RNA virus, SAR-COV-2, both in vivo and in vitro. The toxicity of DTBN was also determined in this paper on Vero E6 cells [21]. These results are consistent with Topoisomerase II being a crucial enzyme associated with replication in parasites [33,34].

DTBN was found to enhance DNA cleavage mediated by human topoisomerase IIα and IIβ. Mechanistic studies with topoisomerase IIα indicate that DTBN is a “covalent poison” that acts by adducting the enzyme outside of the DNA cleavage–ligation active site and requires the N-terminal domain of the protein for its activity [18].

As an immune evasion strategy, *Leishmania* selectively modulates protein kinase PKC-α, PKC-β, PKC-δ, and PKC-ζ isoforms in macrophages to ensure its survival. Selective targeting PKC isoforms using their respective inhibitors in combination significantly modulated anti-leishmanial functions, enabling PKC isoform-targeted anti-leishmanial therapy [35].

DTBN is an effective inhibitor of the kinase activity of members of the PKC family. Molecular docking analysis demonstrated the interaction of DTBN, with the kinase domain of PKCs depicting the best affinity towards conventional PKCs by our in vitro kinase activity data [19].

Cysteine proteases are omnipresent enzymes that are critically implicated in the pathogenesis of protozoic infections. Despite their significance, cysteine proteases as therapeutic targets have not yet been translated into clinical practice [36,37]. Both parasite and host proteinases are involved in the clinical manifestation of leishmaniasis [38].

We have previously demonstrated the specificity of inhibition by DTBN on cysteine proteases. Cathepsin S was the most sensitive to inhibition by DTBN compared to Cathepsin B, L, and papain [20].

Thus, these and other data indicate that DTBN modulates key cellular signal transduction pathways that may be relevant to disease, possibly including leishmaniasis.

In summary, DTBN belongs to a family of compounds with potential anti-leishmanial therapeutic potential [39]. The combined, pleiotropic effects of DTBN on tested target molecules in a variety of cells and pathogens, including *Leishmania*, make it an effective therapeutic agent candidate by itself or in combination with established treatment modalities.

## Data Availability

The original contributions presented in the study are included in the article and further inquiries can be directed to the corresponding author.
